# Between traditions and globalization: value orientations of Kazakhstani youth

**DOI:** 10.3389/fsoc.2025.1563274

**Published:** 2025-04-28

**Authors:** Yermek Buribayev, Zhanna Khamzina, Amina Buribayeva

**Affiliations:** ^1^Zhetysu University named after I. Zhansugurov, Taldykorgan, Kazakhstan; ^2^Department of Law, Abai University, Almaty, Kazakhstan; ^3^Nazarbayev Intellectual Schools, Astana, Kazakhstan

**Keywords:** cultural value orientation, societal value priorities, moral state index, traditional values and moral-ethical concepts, public consciousness

## Abstract

**Introduction:**

In this manuscript, a study of the social values of Kazakhstani youth is presented in the context of the new social reality of independent Kazakhstan. The purpose of this work is to identify the similarities and differences in the value priorities of youth compared to national trends, as well as to determine the factors influencing the formation of values in modern Kazakhstani society.

**Methods:**

The methodological basis of the study is a sociological survey conducted among Kazakhstani youth, whose results were compared with the 2018 World Values Survey (WVS) data. Such a comparative analysis enables a deeper understanding of the specific characteristics of the value orientations of the younger generation.

**Results:**

The value orientations of Kazakhstani youth were analyzed and compared with the results of the 2018 WVS conducted in Kazakhstan. The identified trends reflect the influence of globalization and socio-economic changes on the value priorities of youth.

**Discussion:**

The findings have practical significance for the development of effective youth policies that take into account the specific value orientations and needs of the younger generation. This approach contributes to their social integration and supports the sustainable development of Kazakhstan.

## Introduction

In the three decades since gaining independence in 1991, Kazakhstan has experienced rapid socio-economic modernization that has profoundly affected its social values. The country’s population has grown to around 20 million people as of 2024, with over 60% now living in urban areas (Statistic, 2024). Kazakhstan is ethnically diverse (about 71% Kazakh, 15% Russian, among more than 100 ethnic groups), and it transitioned to an upper-middle-income economy by 2006 after significant oil-driven growth. These transformations have catalyzed a reexamination of societal values: Soviet-era norms have been reassessed, and there is an ongoing revival of national cultural values alongside the adoption of global cultural influences. Notably, the government’s Rukhani Zhangyru (“Spiritual Revival”) program launched in 2017 explicitly aimed to strengthen Kazakhstan’s historical traditions and values while modernizing the national identity. This context of change has led to the emergence of new social paradigms and a search for balance between traditional and modern orientations in Kazakhstani society.

The ongoing transformation of values in Kazakhstan is a complex, multifaceted process that intertwines the legacy of collectivist Soviet ideals with the pressures of globalization and individualism. Social and cultural values constitute a fundamental part of the social structure, guiding behavioral norms and shaping both collective and personal identities (Zharkynbekova et al., 2025). As such, shifts in the value system have significant implications: the ways people prioritize family, religion, work, and self-expression can influence social cohesion and development. Kazakhstan’s case is particularly intriguing because of the palpable tension between longstanding communal values and newer liberal values. For example, recent studies indicate that Kazakhstani youth strongly value close family ties and community, yet at the same time they increasingly aspire to personal success, self-reliance, and career achievement (Salikzhanov, 2014). This duality—evident in the coexistence of collectivist and individualistic value orientations—can create challenges in the socialization of young people, who must navigate between respecting traditional expectations and embracing modern opportunities. Understanding how such value contradictions are negotiated is essential, especially given theories of cultural change which posit that economic development tends to shift societal priorities from traditional, materialist concerns toward more individualistic and “post-materialist” goals (Trastulli, 2021). A comprehensive scientific analysis is therefore needed to capture the dynamics of value change in Kazakhstan’s rapidly evolving society.

Young people are at the forefront of these cultural and value shifts, making the study of youth value orientations especially critical. Youth (defined officially in Kazakhstan as ages 14–29) comprise over one-fifth of the population and act not only as objects of socialization but also as agents of change who will shape the country’s future. This generation has come of age during a period of intensive globalization, urbanization, and digital transformation, all of which expose them to new ideas and lifestyles. Consequently, understanding the evolving values of Kazakhstani youth is increasingly relevant in today’s world (Karipbayev, 2021). Their attitudes and worldviews can provide insight into broader societal trends and potential future developments. Initial observations suggest that while young people continue to uphold core traditional values such as family solidarity and the importance of health, they place greater emphasis on material well-being and personal fulfillment than older generations did (Tkacheva and Baimukhametova, 2016). These emerging value priorities — which include higher expectations for career growth, education, and self-expression — reflect the influence of Kazakhstan’s socio-economic changes and global cultural currents.

In light of these considerations, the present study aims to empirically examine the value orientations of Kazakhstani youth within the context of the country’s sociocultural transformation. We focus on student youth in urban settings and compare our findings with national survey data from the World Values Survey (2018) to identify how the younger generation’s values converge with or diverge from overall societal trends. This comparative analysis enables us to pinpoint the specific value priorities of youth and to determine the factors influencing their formation in modern Kazakhstan. We anticipate that the value orientations of young people will differ in notable ways from those of the general population. In particular, it is hypothesized that Kazakhstani youth maintain strong traditional values (e.g., family orientation) while simultaneously exhibiting a heightened emphasis on personal ambition, entertainment/leisure, and self-realization opportunities in comparison to older cohorts. These shifts in priorities are presumed to be driven by the forces of globalization, urban life experiences, and rapid socio-economic development in the country. Furthermore, we explore the notion that youths’ generally optimistic outlook might correspond with higher reported life satisfaction and greater engagement in socially significant activities (such as volunteering or civic initiatives) compared to the broader population. By addressing these questions, the study seeks to contribute a deeper understanding of the ongoing value changes among Kazakhstan’s younger generation. The findings are expected to have practical significance for developing effective youth policies and programs, by ensuring they are aligned with the evolving value orientations and needs of young people. In turn, such alignment can support better social integration of youth and foster the sustainable development of Kazakhstan’s society in the long term.

## Literature review

Research on the values of Kazakhstani youth has expanded significantly since the country’s independence in 1991. Early studies observed that the younger generation was developing new value paradigms through a process of “borrowing” and creative adaptation of foreign cultural elements to local realities. This dynamic process has been described as youth actively reshaping their value orientations in response to rapid social change (Diana, 2021; Junisbai and Junisbai, 2020; Karipbayev, 2021). Overall, the existing scholarship provides a solid foundation for understanding how Kazakhstan’s post-Soviet transition affected youth values. For instance, Kenzhebayeva et al. (2018) highlight an “education effect” in value formation: higher educational attainment among youth correlates with shifts in life priorities and career aspirations. In other words, university-educated young people tend to develop different value priorities (such as greater emphasis on self-development and professional growth) compared to those with less education. These findings underscore that youth values in Kazakhstan are not static; they evolve with changing socio-economic conditions and personal opportunities.

Contemporary studies consistently report a duality in the value orientations of Kazakhstani youth. On one hand, core traditional values remain strong. Family, in particular, continues to hold paramount importance for most young people. Surveys find that the vast majority of youth maintain close family ties and express high trust in their parents and relatives (Laruelle, 2019). The Youth in Kazakhstan national study (2021) concluded that young people “tend to have more traditional attitudes toward family, marriage, and children,” reflecting continuity of cultural norms (Sharipova and Bejmenbetov, 2021). At the same time, Kazakhstani youth are increasingly drawn to individual success and self-realization. Researchers have observed that while young Kazakhs deeply value their families and communities, they “aspire to personal success, self-reliance, and career achievement” to a greater extent than previous generations. This coexistence of collectivist orientations (e.g., family solidarity) with emergent individualistic goals (ambition, career advancement) has been documented in multiple studies. For example, Salikzhanov’s survey (Salikzhanov, 2014). of urban youth found that strong filial piety and community loyalty went hand-in-hand with a heightened desire for economic independence and higher living standards among today’s youth. Value shifts in Kazakhstani society have been extensively studied by scholars such as Dave (1996), Shormanbayeva et al. (2014), Beissenova et al. (2014), and Kilybayeva et al. (2017). These researchers conceptualized the emergence of new value paradigms as processes of “borrowing” and “creative adaptation” to a changing social environment. This approach emphasizes the dynamic nature of youth value orientations and their capacity to adapt to rapid social transformations. Thus, the literature indicates that Kazakhstani youth navigate a nuanced mix of values: they uphold many traditional norms even as they adopt certain modern values centered on personal achievement.

Kazakhstani society’s rapid modernization over the past three decades provides essential context for understanding youth values. The country’s swift urbanization, economic growth, and nation-building efforts have “catalyzed a reexamination of societal values,” including a revival of national cultural values alongside the influx of global ideas. This has created a social environment in which young people are pulled between tradition and modernity. On the one hand, Kazakhstani youth inherit the legacy of Soviet-era collectivism and indigenous cultural norms; on the other hand, they face the pressures of an increasingly individualistic and globalized lifestyle. Zharkynbekova et al. (2025) describe this ongoing transformation of values as a complex interplay between longstanding communal values and newer liberal influences. In particular, many youths experience a palpable tension between respecting traditional expectations (e.g., obedience to elders, collective interests) and embracing the liberal ethos of personal freedom and self-expression.

Empirical research suggests that Kazakhstan’s younger generation is indeed undergoing value change in line with modernization theory. According to Inglehart’s modernization framework, economic development tends to shift social values from survival and tradition toward greater individual choice and self-expression. In Kazakhstan’s case, studies have found evidence of rising individualism among youth born in the post-Soviet era. Karipbayev’s analysis of student youth, for example, reveals a growing trend of individualism and indifference to traditional forms of identity in this cohort. Young people appear less constrained by old collective identities tied to clan, ethnicity, or strict gender roles, compared to their parents’ generation (Karipbayev, 2021; Junisbai and Junisbai, 2020) likewise report generational shifts in attitudes: the so-called “Nazarbayev generation” (those who came of age after 1991) is more accepting of socioeconomic inequality and places greater faith in self-reliance, rather than expecting state support or communal help as the older generation might have. This finding points to an embrace of market-oriented values and personal responsibility among today’s youth. Notably, while Kazakhstani young people still broadly respect their elders and cultural traditions, they do not necessarily adhere to all traditional prescriptions. For instance, they may prioritize education and career over early marriage, or value meritocratic success over family connections, to a greater degree than was common a generation ago. Overall, the literature indicates that the forces of modernization – urban living, expanded education, economic diversification, and exposure to new ideas – have begun to recalibrate the value system of youth, fostering more individualistic outlooks even as some traditional anchors persist.

In tandem with internal modernization, the impact of globalization has become a central theme in studies of Kazakhstani youth values. Globalization has connected Kazakhstan’s youth with worldwide cultural currents, whether through the internet, social media, global pop culture, or the diffusion of international norms. Scholars note that Kazakhstan is one of the most globally integrated of the Central Asian states, which has accelerated the spread of global values among its population (Starr et al., 2016; Batsaikhan and Dabrowski, 2017). Karipbayev (2021) observes that contemporary Kazakhstani youth live amid “global changes in the value system and the simultaneous revival of traditional values,” a combination that can produce uncertainty in their self-identity. On the one hand, young people are drawn to universal ideas of individual rights, consumer culture, and technological savvy that globalization brings; on the other hand, they are also witnessing a resurgence of interest in national heritage, language, and religion (sometimes termed “neotraditionalism”) as a counterbalance to globalization. This dual influence forces youth to negotiate between global and local value frameworks. Recent studies (Sharipova, 2020; Zharkynbekova et al., 2025) highlight that young Kazakhs often strive to blend these influences rather than choosing one over the other. For example, in surveys many urban youths’ express pride in Kazakh cultural traditions even as they adopt cosmopolitan lifestyles and global media preferences. They might celebrate traditional holidays and family customs, yet also support global movements for environmentalism or gender equality.

Researchers emphasize that understanding this interplay of global and local forces is crucial for interpreting youth values in Kazakhstan (Mussabekov, 2024; Rakisheva et al., 2024). Some have framed the identity choices of young Kazakhstanis as a spectrum: at one end, a youth identity anchored in ethnic culture and tradition; at the other end, an identity aligned with global (or Westernized) values of individuality and openness. In practice, most youth fall somewhere in between, constructing hybrid identities that draw from both sources (Dussipova et al., 2025). For instance, qualitative interviews in one study found that students often spoke of balancing their “Kazakhness” (respect for elders, community obligation) with being a “world citizen” who values diversity and personal autonomy. This balancing act reflects globalization’s nuanced impact: it does not erase local culture but rather interacts with it, sometimes reinforcing certain traditions even as it undermines others. Indeed, Kazakhstan’s government has itself promoted a blend of global and traditional values – a policy exemplified by the Rukhani Zhangyru (Spiritual Revival) program that seeks to modernize the national identity while preserving cultural heritage (Aimaganbetova et al., 2024).

Empirical data bear out the complex effects of globalization on youth attitudes. Large-scale surveys have found that Kazakhstani youth hold a mix of outlooks in line with global trends as well as local specificities. For example, young people overwhelmingly endorse the importance of obtaining a quality education and building a successful career, aspirations common to youth worldwide in an era of global competitiveness (Laruelle, 2019). Many also voice support for universal values like human rights and equality – albeit “to some extent,” as one study carefully notes – indicating a cautious embrace of liberal democratic ideals. At the same time, Kazakhstani youth display distinctly local attitudes on social and moral issues; they tend to be socially conservative by Western standards, expressing reservations about topics like premarital sex or LGBTQ+ rights. This suggests that global liberal norms have only partially penetrated the private value sphere, where traditional norms (often informed by Kazakh culture and religion) still prevail (Sharipova and Bejmenbetov, 2021). In short, globalization has opened Kazakhstani youth to new values and lifestyles, but these influences are continually mediated by the country’s cultural context.

Taken together, prior studies paint a picture of a generation negotiating a delicate balance between old and new value systems. Culture remains a powerful force: familialism, community orientation, and respect for tradition are deeply ingrained in youth upbringing and continue to shape their worldview. At the same time, the forces of modernization – increased education, urbanization, and economic change – have introduced more individualistic and materialist values, evident in youths’ rising emphasis on personal ambition and self-reliance (Junisbai and Junisbai, 2020). Moreover, ongoing globalization exposes young Kazakhstanis to worldwide currents, from pop culture to political ideas, resulting in a generation that is outward-looking and adaptive but not wholly detaching from its roots. This complex interplay of value orientations, cultural tradition, modernization, and globalization is at the heart of current sociological discourse on Kazakhstani youth. Building on these insights, the present study will examine how these dynamics are manifesting among today’s youth, identifying which values are changing and which endure, and what this means for Kazakhstan’s social development. The literature provides a rich context that situates our inquiry within broader debates on value change in post-Soviet and rapidly modernizing societies. Our research aims to contribute to this body of knowledge by offering up-to-date empirical evidence on the value priorities of Kazakhstani youth and the factors influencing them in the current era.

## Methodology

This study employed a comparative cross-sectional design that integrates both secondary data analysis and primary quantitative research to examine the value orientations of Kazakhstani youth. An interdisciplinary theoretical framework was adopted, bridging philosophical and sociological approaches to the problem of value formation. In particular, the study is grounded in an axiological approach – focusing on the nature and hierarchy of values – and utilizes systems thinking to consider values within the broader social system of Kazakhstan. A hermeneutic method provided an interpretive lens for understanding the cultural meaning behind observed value preferences. Additionally, comparative and retrospective analyses were applied, enabling us to juxtapose the values of young people against wider societal trends and to consider historical shifts. This design allowed us to connect theoretical insights on value change with empirical data, combining a secondary analysis of existing survey data with new evidence gathered specifically for this research. The result is a holistic view of youth value orientations in the context of Kazakhstan’s ongoing socio-cultural transformation.

The primary data for this study came from a structured survey conducted among university students in Kazakhstan’s two largest urban centers, Almaty and Astana. A total of 600 respondents (346 women and 254 men) between the ages of 18 and 23 participated in the survey, which was carried out in October–November 2024. Participants were recruited from local universities, targeting the student youth population as a critical subgroup for examining emerging values. The survey was administered through face-to-face interviews by trained field specialists, with respondents completing the questionnaire in their language of preference (Kazakh or Russian). This personal, in-person data collection approach yielded a very high participation rate – over 90% of those approached agreed to take part – indicating excellent respondent engagement and minimizing nonresponse bias. All respondents participated voluntarily with assurances of confidentiality.

In addition to the new survey data, the study incorporated secondary data from the World Values Survey (WVS). Specifically, we used the Kazakhstan dataset from WVS Wave 7 (collected in 2018) as a point of reference for national value orientations. The WVS is a globally recognized research program that provides nationally representative data on public beliefs, values, and attitudes; the Kazakhstan WVS sample offers a broad context against which the values of our urban youth sample can be compared. By including this secondary source, our research design gains a comparative dimension: it situates the value priorities of contemporary student youth vis-à-vis the overall societal values captured by the WVS. (Further details on the WVS sampling and methodology can be found in the official WVS documentation by Haerpfer et al., 2024.) This two-pronged data collection strategy—combining a fresh survey of youth with an analysis of existing WVS data—was intended to illuminate both the unique features of youth values and their alignment with or divergence from wider cultural trends in Kazakhstan.

The survey instrument for the primary study was derived from the standardized questionnaire of the World Values Survey, tailored to focus on key cultural and social values relevant to youth. We selected a subset of questions from the WVS Wave 7 questionnaire that pertain to value orientations in domains such as family and community relations, religion and morality, life goals and well-being, and attitudes toward societal change. These items were chosen because they directly address the core value priorities of interest (e.g., the importance of family, attitudes toward tradition versus modernization, priorities in life such as career, education, and leisure, and other aspects of cultural values). Using the WVS question formulations ensured that each survey item had been previously tested and validated internationally, thereby enhancing the content validity and comparability of our measures. The questionnaire was provided in both Kazakh and Russian, using officially translated versions of the WVS items to ensure linguistic accuracy and clarity for all respondents. Trained interviewers guided participants through the questions in a consistent manner, which helped reduce misunderstandings and maintain data quality.

It is important to note that the survey mainly employed single-item indicators for each value concept, rather than multi-item composite scales. Each variable of interest (for example, “importance of religion in one’s life” or “priority of career success”) was measured with one direct question. Consequently, it was not necessary to compute internal consistency reliability measures such as Cronbach’s alpha for our instrument – such measures are not applicable when only single items are used to represent each construct. Instead, we relied on the established reliability of the WVS items themselves, as evidenced by their repeated use in cross-national research. In summary, the instrument was a vetted questionnaire capturing a range of value orientations, administered in a culturally appropriate and understandable format for the target youth audience. This allowed us to confidently gather data on students’ value priorities with minimal measurement error and strong alignment to the concepts measured in the national WVS.

All collected data from the student survey were compiled and analyzed using SPSS Statistics (Version 12.0) and Microsoft Excel. We began by performing data cleaning and verification, then proceeded with descriptive statistical analysis. Given the aims of the study, the analysis was intentionally limited to descriptive statistics such as frequency distributions, percentages, and mean or median values for the key survey items. These descriptive results illustrate the prevalence and ranking of various values among the respondents. We also examined the data for any notable patterns or differences across subgroups within the sample (e.g., by gender), although the primary emphasis remained on the overall trends in the youth sample.

A comparative analysis was then conducted to place the youth survey findings in context with the 2018 WVS data for Kazakhstan. For each value-related question that our study shared with the WVS, we aligned the results from our student sample with the corresponding national results. This side-by-side comparison allowed us to observe how the young urban respondents’ values converge with or diverge from those of the broader population. It is important to clarify that this comparison with WVS is used in a contextual and exploratory manner; we did not perform formal statistical tests (such as chi-square or t-tests) to assess the significance of differences between our sample and the WVS sample. There are several reasons for this choice. First, the two datasets are not strictly comparable in a statistical sense: our youth sample is a specific subset (urban university students) collected in 2024, whereas the WVS represents a cross-section of the entire Kazakhstan population in 2018. The sampling methods and time frames differ, meaning any direct significance testing could be misleading. Second, our research objective was to identify and discuss patterns and potential differences in value orientations, rather than to make inferential claims about the population at large. Therefore, we treated the WVS figures as a benchmark for contextual understanding rather than as a control or comparison group in a hypothesis-testing framework.

In practice, this means that observed differences or similarities between the youth survey and the WVS data are interpreted qualitatively and with caution. For example, if a particular value (such as the importance of religion or attitudes toward authority) appeared more pronounced among youth than in the general WVS data, we discuss this trend as a noteworthy finding in the context of Kazakhstan’s social change, but we refrain from labeling it a statistically significant difference. By limiting the analysis to descriptive statistics and careful comparative observations, we ensured that the conclusions drawn are directly supported by the data without overextension. All statistical analyses were conducted with oversight to verify accuracy, and results are presented in aggregate form. This analytical approach, while modest, is appropriate for the exploratory nature of the study and provides meaningful insights into how Kazakhstani youth values are positioned between traditions and globalization, in comparison with broader societal values.

## Results

To facilitate a direct comparison of findings, Table 1 presents key value orientations and survey questions from the Kazakhstani Youth Survey alongside analogous results from the 2018 World Values Survey (WVS) in Kazakhstan.

**Table 1:** Key indicators from the Kazakhstani Youth Survey compared to the 2018 World Values Survey (Kazakhstan), including additional value-related items.

Value orientation/Indicator	Youth survey (Youth sample)	WVS 2018 (General population)
Values		
Family – considered an important value	80.1% of youth identified family as their primary value	99.6% of respondents consider family important
Health – considered an important value	63% of youth cited health as a key value	~95% rated health as “very important”
Friendship – importance of friends	37.9% of youth consider friendship important	84.1% of respondents consider friends important
Leisure and entertainment – value of free time	42.0% of youth value leisure and entertainment	78.6% of respondents consider free time important
Materially secure life – value of material well-being	42.4% of youth value a financially secure life	No directly comparable WVS data
Work – importance of an interesting job/profession	22% of youth prioritize having an interesting job	87% of respondents consider work important
Religion – considered an important value	18.4% of young people identified religion as their main value	64.9% of respondents consider religion important
Environmental protection – prioritized even at economic cost	~53% of youth favor protecting the environment over economic growth	~44% of respondents favor environmental protection over growth
Well-being		
Life satisfaction – share of respondents who are “satisfied” with life	73.4% of youth satisfied with their life overall	42.3% of general population satisfied with life
Self-assessed financial well-being – the proportion of respondents reporting their financial situation as “satisfactory”	49.8%	27,1%
Self-rated health – share reporting “good” or “very good” health	84.7% of youth rate their health positively	68.2% of general population rate their health positively
Institutional trust		
Trust in government – share who trust government	22% of youth trust government officials	~68% of respondents trust government officials
Trust in political parties – share who trust parties	14.0%	57.0%
Trust in mass media – share who trust mass media	15.4%	61.3%
Overall institutional trust index (composite trust level)	18.0%	62.1%
Participation in volunteer activities – share engaged in volunteering	34% of youth have participated in volunteer activities	9% of respondents engaged in volunteer work
Support for protest activity – share supporting/considering protest as civic action	38.7% consider participation in permitted protest actions effective	~24% of respondents have done or would join a peaceful protest
Level of religiosity – self-identified believers vs. non-religious vs. atheists^3^	Believers: ~72%; Not religious: ~23%; Atheists: ~5%^3^	Believers: ~80%; Not religious: ~15%; Atheists: ~5%

Youth Survey refers to the authors’ 2024 survey of urban Kazakhstani youth (ages 18–23, *N* = 600). WVS 2018 refers to the Kazakhstan sample of the World Values Survey Wave 7 (2018). All figures are the percentage of respondents endorsing each item (e.g., considering the value “important,” reporting being “satisfied,” or expressing trust). “Overall institutional trust index” is a composite measure of confidence in institutions, calculated here as the mean of the trust in government and trust in political parties (the two institutional trust indicators measured in both surveys).

The comparative overview in Table 1 highlights both notable similarities and differences between Kazakhstani youth and the general population in terms of values and well-being. Family clearly stands out as the dominant value in both groups, with an almost universal importance in the WVS data (nearly 100% of respondents) and a very high priority among youth (80%). This consistency underscores that strong familial orientation remains a cornerstone of Kazakhstani society across generations. Both sources also indicate appreciation for leisure time and personal well-being, though these aspects show differing levels of emphasis: the general population places greater importance on leisure (nearly 79% in WVS) than the youth do on entertainment (42%), suggesting a possible difference in how free time is valued or how the questions were framed. It is noteworthy that the youth survey shows that health is the most important personal value for young people (63% mention health), which is in line with global trends, where young people prioritize well-being. According to the 7th wave of the World Values Survey (2017–2022), family remains the priority life value for Kazakhstanis, but health almost equally shares the top of their value system. The overwhelming majority of respondents in Kazakhstan consider health to be one of the most important aspects of life (about 95% indicated it as “very important”), that is, almost as unanimously as in relation to family.

This hierarchy of values is in line with global trends: everywhere people put family well-being and good health first, while slightly less importance is attached, on average, to, for example, work, friends or leisure. Compared to other countries, the role of health in the values of Kazakhstanis is equally high – health is consistently among the top priorities, similar to observations in many societies around the world.

Overall, both surveys confirm that Kazakhstani respondents – young and old – share some core values (especially family), but differ in the ranking and visibility of certain personal values.

At the same time, the table illustrates significant differences in value orientation between the younger generation and the broader population. Kazakhstani youth place considerably less emphasis on friendship and work as paramount values compared to the general population: for example, only 38% of youth consider friends important, versus 84% in the WVS sample, and a mere 22% of youth prioritize work ambition, compared to 87% in the national data. These gaps may reflect generational shifts toward individualism or life-stage effects—young people might currently be more focused on personal development and self-expression than on work or expansive social circles. The importance of religion also varies: while around 65% of the general population consider religion important (WVS 2018), in the youth survey only 18.4% of respondents said that religion had the greatest value in their lives, which may indicate that organized religion plays a less central or more private role in young people’s values. In terms of subjective well-being, the findings show that life satisfaction is markedly higher among youth: roughly three-quarters of young respondents’ report being satisfied with their lives, in contrast to about 40 % of the general population. Likewise, self-rated health is substantially better among youth, with 85% of young people describing their health as good or very good, versus 68% in the broader sample—an expected difference given the relative youth and physical advantages of the younger cohort. These contrasts in life satisfaction and health perceptions suggest that younger Kazakhstani people are more optimistic about their present circumstances, potentially due to better health and future outlook, whereas older respondents (making up much of the WVS sample) may face more life challenges tempering their satisfaction.

In summary, the Youth Survey and WVS 2018 data together paint a picture of a younger generation that both shares and departs from the values of the wider society. Kazakhstani youth uphold the primacy of family bonds much like their elders, and they demonstrate concern for personal well-being and enjoyment, yet they exhibit a lower attachment to certain traditional or collective values such as long-term work commitment, friendship networks, and possibly religious observance. Simultaneously, young people report higher personal well-being (in terms of life satisfaction and health), highlighting a generational divergence in outlook. These differences and similarities are crucial for understanding how social values are evolving: they indicate that while foundational values persist across generations, Kazakhstani youth are navigating a shift toward more individual-centered priorities and optimism. This insight can inform policymakers and educators as they seek to engage the youth demographic, suggesting the need to acknowledge the changing value landscape—such as the emphasis on health and personal autonomy—while also addressing areas where youth may be less connected to traditional societal structures (e.g., formal religious or professional life). Overall, integrating the youth perspective with national trends is essential for developing social programs that resonate with young people’s values and for ensuring that the enduring importance of family and community is harnessed to support the country’s future development.

## Discussion

The discussion is organized as a comparative analysis of the Youth Survey results and the 2018 World Values Survey (WVS 2018) data. It is divided into two subsections: the first highlights the similarities in value orientations between the youth cohort and the general population, and the second examines the differences between these groups. Organizing the discussion around this youth vs. general population comparison allows for the identification of both enduring cultural elements (values that remain consistent across generations) and signs of an intergenerational shift in values. Such a comparative approach is well-founded in the scholarly literature on intergenerational value change, which emphasizes that cohort comparisons can reveal stable value continuities as well as emerging shifts in societal priorities (Henderson, 2024; Inglehart, 2016). By juxtaposing the congruences and divergences in values between younger and older generations, this approach sheds light on the extent of cultural continuity versus change, helping to pinpoint which values persist as robust norms and which may be evolving in a generational transition.

### Findings aligned between the two surveys

The comparative analysis reveals several value orientations that Kazakhstani youth share with the broader population, indicating continuity across generations. Family stands out as a paramount value in both groups, affirming that strong familial bonds remain a cornerstone of Kazakhstani society. In our youth survey, 80% of respondents identified family as their primary value, and in the 2018 WVS nearly all respondents (about 99%) likewise rated family as “very important” in their lives. This convergence underscores enduring loyalty to the family unit as a fundamental priority, reflecting the persistence of a traditional collectivist ethos despite rapid social change. The primacy of family across young and old is consistent with prior research emphasizing that some traditional anchors persist even amid modernization. It suggests that, regardless of generation, Kazakhstani individuals continue to derive identity, support, and meaning from close family relationships – a trait deeply rooted in the cultural fabric.

Beyond family, both youth and the general population express concern for personal well-being and quality of life, though the emphasis varies. For instance, the surveys indicate that leisure or free time is valued by both cohorts, pointing to a common appreciation for work-life balance and enjoyment of life. In the WVS data, about 79% of Kazakhstani adults considered leisure time important, and similarly a notable share (42%) of youth in our study valued entertainment and free time. While the absolute percentages differ, the presence of leisure as a valued domain in both samples suggests a generational continuum in seeking personal fulfillment beyond mere material survival. Both groups also recognize the importance of material security and health, again to varying degrees of importance. A significant minority of young people (42.4%) value living financially securely, implying that economic well-being is a recognized goal for young people as it is for older people.

Health is also emerging as a prominent priority for young people – 63% cited good health as key – and 95% selected health as an important priority in the 2018 WVS. In fact, the vast majority of young respondents (85%) in our survey described their health as good or very good, and a significant majority of adults in the WVS (68%) also reported positive health. These results suggest that both younger and older generations of Kazakhstanis prioritize basic well-being needs – having a healthy, secure life and time for personal enjoyment – alongside their shared priority of family. Such commonalities echo elements of modernization theory, which posits that as societies develop, people increasingly emphasize quality of life values in addition to traditional values. In the case of Kazakhstan, the data suggest that core values such as family solidarity coexist with emerging aspirations for personal well-being across generations, illustrating the combination of tradition and modernity in the value system.

It should also be noted that high levels of self-rated health among young people are an expected outcome of their age, but this finding is subject to interpretation. In general, good health among young people can be seen as a baseline condition that allows other value orientations to flourish. In our study, young people not only report better health than the older population (as expected), but they also tend to be more optimistic about life in general—about 73% of young people are satisfied with their lives, compared to 42% of the general population. This higher life satisfaction and confidence likely stems from the youth’s physical strength and future prospects, confirming what the reviewer noted: good health in early life provides a foundation for pursuing higher-order goals such as educational ambitions, career advancement, and self-actualization. In other words, because most young people feel physically well, they may take health for granted and focus more on personal growth and self-expression. This insight is consistent with classical theories of human development, which suggest that once basic needs (e.g., health) are met, people can shift their focus to personal achievement and self-actualization. Our findings support this idea: with largely secure health, many young people in Kazakhstan focus on self-development and enjoyment values, as evidenced by their interest in leisure activities and relative optimism about life.

Thus, the shared importance of well-being among youth and adults’ highlights continuity in valuing fundamental quality of life, even as the better health and optimism of younger generations prepares them to place greater emphasis on self-actualization. In summary, the converging results of the youth survey and the 2018 WVS indicate that Kazakhstani youth are not in complete opposition to the values of their elders. Fundamental priorities—especially family loyalty and basic well-being—are common threads that unite generations. These commonalities reflect how cultural traditions (such as family loyalty) remain deeply ingrained, while the benefits of modernization (better health, more leisure time, and economic security) are widely valued by both young and old. This continuity provides a stable social foundation on which differences in other values are layered. It also suggests that efforts by families, educators, or policymakers to engage young people can build on these shared values of family and well-being as a bridge between generations. In particular, maintaining family-centered values provides a point of social cohesion that can help mitigate potential ruptures arising from other divergent values discussed below.

### Findings that differ between the two surveys

Despite these common threads, our study also uncovers pronounced divergences in value orientations between Kazakhstani youth and the general population, highlighting the generational shift underway. Young people in our survey prioritize certain values much less than their elders do, pointing to a reordering of importance that reflects forces of secularization, individualization, and the unique life-stage of youth. One of the most striking differences is in attitudes toward social relationships and work. The youth sample places considerably lower emphasis on friendship and work compared to the WVS adult sample. For example, only 38% of youth deemed friendship to be very important in their lives, whereas 84% of the general population reported friends as important. Similarly, a mere 22% of youth said having an interesting job or career is a top priority, in contrast to 87% of adults who consider work important. These gaps are substantial. They may partly reflect a life-stage effect – many of our respondents are university students whose immediate concerns might center on education and personal exploration rather than career or widening their social circle. However, the magnitude of the differences also suggests a broader generational shift toward individualism and self-reliance. The lower emphasis on friendship networks and formal work roles could indicate that today’s youth place relatively more value on individual pursuits and intimate family ties, rather than on broader social or organizational commitments. In other words, young Kazakhs seem to be orienting their lives less around external structures (like workplace identity or large peer groups) and more around personal goals and close-knit relationships. This interpretation finds support in previous studies: Karipbayev (2021) observes a growing “indifference to traditional forms of identity” among student youth – for instance, less attachment to communal or collective identities than their parents had. Our findings echo this trend of individualization, where the younger generation feels less bound by some of the social expectations that defined earlier generations.

Another key divergence is in the realm of religious values and secularization. Religion remains an important part of the older generation’s value system – about 65% of Kazakhstani adults in 2018 said religion is important in their lives. In contrast, our youth survey did not find religion ranking among top values for young people. The absence of religion as a prioritized value for the youth, juxtaposed with its significance in the WVS data, suggests that young Kazakhstani respondents are generally more secular in orientation. This generational difference aligns with the broader secularization thesis that modernization and improved existential security tend to diminish the centrality of religion.

Scholars (Spehr and Kassenova, 2012; Karimov et al., 2024) have noted that post-Soviet Kazakhstan has seen a revival of interest in religion and national traditions among parts of the population (sometimes termed “neotraditionalism”). However, the youth in our study, having grown up in an era of relative stability and global cultural exposure, appear less influenced by this religious resurgence. Their value priorities lean more toward this-worldly concerns (education, personal success, enjoyment) than toward spiritual or faith-based ones. In essence, the younger generation is more secular and individual-centric, whereas the older generation retains stronger religious attachments as part of their value mix. This does not necessarily mean young people are irreligious, but rather that faith and church/mosque participation likely occupy a more private or peripheral role in their lives, compared to the explicit weight it holds for many older adults. Our findings here resonate with Norris and Inglehart’s observations on secularization: as societies modernize and younger cohorts grow up with greater sense of security, religious values often recede in importance (Norris and Inglehart, 2011). The Kazakhstani case seems to follow this pattern, with youth exhibiting a tilt toward secular, individual values in lieu of overt religiosity.

Perhaps the most consequential disparities revealed by our study lie in the domain of social and institutional trust. Our survey included items on trust in various institutions and public figures, and the contrasts with WVS data are stark. Kazakhstani youth report significantly lower trust in institutions and leaders than the general population does. For example, only about a quarter of youth in our sample expressed trust in government officials, whereas in the 2018 national WVS over two-thirds of respondents (around 68%) said they trust the government. Trust in political parties shows an even wider gulf: merely 14% of youth trust party leaders, versus 57% of adults in WVS. A similar pattern emerged for trust in the media and experts – our young respondents were skeptical of traditional news sources, even while WVS indicates majority of Kazakhstani adults trust mass media to some degree. These findings point to a high level of social distrust or critical skepticism among the youth, especially toward formal institutions and authority figures.

In contrast, the older generation (as captured in 2018) appeared relatively more trusting of state institutions and mass media. This divergence in trust is highly relevant to the discussion of values: it suggests that young people today approach society with a more questioning, less deferential attitude than the previous generation. The youth’s low trust can be interpreted through the lens of global trends in social capital – consistent with observations by Putnam and others that younger generations in many societies are less engaged in civic institutions and less trusting of authorities than were their predecessors at the same age (Putnam, 2000).

In Kazakhstan’s context, one might attribute this to several factors. Post-independence youth have grown up in an era of information pluralism (and misinformation) via the internet, exposure to global media, and awareness of corruption or inequalities, which may foster cynicism about official narratives and public institutions. Moreover, as Junisbai and Junisbai (2020) note, the “Nazarbayev generation” places greater faith in self-reliance than in state support. Our results strongly echo that point: young people seem to trust mainly themselves and perhaps their immediate circles, rather than formal organizations or distant leaders. This could be seen as a decline in public loyalty – loyalty in the sense of allegiance to state institutions or collective enterprises – among the youth cohort. Instead of identifying with large-scale institutions (parties, government, religious authorities), youth may be channeling loyalty to more personal spheres (family, close friends) or abstract ideals of self-achievement. Indeed, previous research in Kazakhstan found that youth tend to express the highest trust in their family members, while almost no one beyond those circles is trusted fully. Such a selective trust pattern indicates that bonds of loyalty are confined to the private domain for many young people, whereas generalized social trust is fragile. This is a pivotal difference from older generations, who, perhaps shaped by the Soviet legacy of collective structures or the early independence years’ nation-building, show comparatively higher trust in public institutions.

On environmental issues, Kazakhstani youth demonstrate a cautious yet noticeable shift toward greater ecological awareness in comparison to the national baseline. The youth survey reveals a nuanced environmental stance: young respondents generally acknowledge the importance of protecting the environment and voice moderate support for sustainable initiatives (for example, many express approval of developing renewable energy sources as part of Kazakhstan’s future). These pro-environment inclinations, however, are tempered by pragmatic considerations. Unlike some ardently “green” youth movements elsewhere, Kazakhstan’s young generation tends to balance ecological ideals with economic realities. For instance, while they favor cleaner energy and conservation in principle, they remain mindful of the country’s development needs, showing ambivalence about sacrificing economic growth for environmental goals. Indeed, the survey found gaps in climate-change awareness among youth: global issues like climate change and global warming are not fully on their radar. Opinions on the severity of global warming appear mixed, suggesting that a portion of youth have not internalized or are skeptical of the urgency of this issue. This partial awareness implies that environmental concern among Kazakhstani youth is often localized—focused on immediate pollution or resource issues—rather than rooted in the broader climate crisis narrative.

By contrast, the 2018 WVS data at the societal level indicate that Kazakhstan’s general population remains predominantly oriented toward economic growth over environmental protection. Nationally, values still skew toward “survival” priorities, with environmental protection taking a backseat to material well-being (Kazakhstan’s cultural profile in WVS falls in the survival-values spectrum, a zone typically marked by lower emphasis on post-materialist concerns like ecology). In this context, youth stand out subtly: they exhibit greater inclination toward environmental values than their elders, even if not to a dramatic degree. The contrast suggests an incipient generational shift—young people are beginning to integrate self-expression values such as environmental consciousness into their worldview, in ways largely absent among the older generation. Still, this shift is nascent and measured. Youth attitudes reflect a both-and approach: a willingness to consider green priorities alongside economic ones, rather than an outright transformation of values. In academic terms, one could interpret this as evidence of gradual movement toward post-materialist values among Kazakhstani youth, tempered by the country’s developmental context. The younger generation’s moderate environmentalism, compared to the wider society’s growth-first mindset, highlights a meaningful but evolving divergence. As educational exposure and global connectivity increase, Kazakhstani youth may further close the awareness gap on climate change and strengthen their commitment to environmental protection. For now, however, their values straddle traditional economic pragmatism and emerging ecological concern, marking a transitional stage in which environmental attitudes are growing in importance but remain balanced by pressing socio-economic priorities.

The youth survey data reveal that young people in Kazakhstan largely navigate modest financial circumstances. Nearly half of the respondents (about 49.8%) characterize their personal finances as only sufficient for basic needs like food and clothing, falling short of what would be required for major purchases such as real estate or automobiles. Correspondingly, their spending is concentrated on essentials: for example, roughly 77% report food as a primary expense and about 66.7% prioritize spending on clothing and footwear. This spending pattern indicates limited discretionary income, as most resources are devoted to immediate necessities. Notably, a small but significant subset of youth (4.2%) live in conditions of severe material hardship, illustrating the presence of a vulnerable group facing acute financial constraints within the younger population.

When these youth perspectives are compared to broader national data, an intriguing generational contrast in financial satisfaction emerges. According to the 2018 World Values Survey, only 27.1% of Kazakhstan’s overall population reported being satisfied with their financial situation—a proportion that appears lower than self-reported contentment levels among youth. This discrepancy can be interpreted through a life-cycle effect and a sense of generational optimism. Younger individuals, despite objectively limited means, often maintain higher optimism and lower immediate expectations regarding their finances, anticipating improved earnings and living standards as they progress through careers and adulthood. In other words, youth may view their current financial limitations as a temporary stage of life, whereas older adults—being at a later life stage—tend to evaluate their financial satisfaction more critically against long-held aspirations. This generational outlook helps explain why young people report relatively higher satisfaction under conditions that older cohorts find less acceptable.

At the same time, the data underscore that optimism among youth exists alongside tangible economic constraints. The fact that nearly half of young respondents lack the means for major investments (such as purchasing property or vehicles) highlights enduring economic barriers that could impede their upward mobility and long-term socio-economic development. The high proportion of expenditure devoted to basic needs further suggests that many youths have limited capacity to save or invest in areas like education, health, or personal development—factors that are important for human capital accumulation over the life course. Additionally, the existence of a vulnerable minority of youth (4.2% in severe financial distress) points to persistent inequalities and the risk of social exclusion within the younger generation. Overall, while Kazakhstani youth exhibit a form of resilience or optimism consistent with their stage in the life cycle, their reported financial well-being must be understood in the context of broader structural challenges, as reflected in the generally low financial satisfaction observed across the country’s population.

As noted, young respondents report far higher life satisfaction than the general population, which can be interpreted in multiple ways. It may be a life-cycle phenomenon – youth generally have optimistic outlooks before the realities of middle age set in – but it also reflects the fact that today’s youth came of age during a period of economic growth and expanding opportunities in Kazakhstan (the 2000s and 2010s), whereas the older population includes those who lived through the hardship of the 1990s transition. The nearly three-quarters of youth who are satisfied with life (versus about two-fifths of adults) might signify a generation imbued with confidence about the future and personal agency. This optimism correlates with their better health and perhaps with greater educational attainment or global connectivity. By contrast, older individuals – some of whom experienced instability or still face economic insecurity – register lower satisfaction. From a values perspective, the greater optimism and well-being of youth could reinforce their tilt toward self-expression values, as theorized by Inglehart. When basic needs (safety, health, income prospects) are met, people tend to prioritize self-expression, autonomy, and happiness more strongly. Our youth sample fits this profile of a post-materialist shift: relatively secure and healthy, they are more inclined to value personal fulfillment (e.g., leisure, self-realization) and less inclined to emphasize survival-oriented values (e.g., needing strong social support networks or job security at all costs). In contrast, the general population’s lower life satisfaction might keep their focus on more pragmatic or survival values (e.g., holding a stable job, relying on community/friends), as suggested by their high emphasis on work and friendships for support. Thus, the well-being gap is both a consequence of age and an enabler of further cultural change – it provides the conditions for youth to depart even more from the traditional mindset.

These divergent findings point to a complex sociological portrait of Kazakhstani youth: they are navigating a new value landscape that departs in important ways from that of their parents. Several theoretical frameworks help make sense of these shifts. Modernization theory offers one explanation: as Kazakhstan has modernized, younger generations have grown up with greater material security and global exposure, fostering a move from collectivist, survival values toward more individualist, self-expressive values. Our evidence of youths’ weaker emphasis on institutional religion, their lower deference to authority, and their focus on personal life satisfaction all correspond to this modernization-driven value change. Inglehart’s classic thesis of intergenerational value change – a shift from “Survival” values (emphasizing economic and physical security, conformity, authority) to “Self-Expression” values (emphasizing autonomy, subjective well-being, and quality of life) – is clearly manifested in Kazakhstan’s case. Young people, having not experienced the deprivations of the past, exhibit more postmaterialist leanings (e.g., prioritizing self-realization over economic or religious duties) than the older cohort.

Additionally, the theory of cultural globalization is pertinent: Kazakhstan’s youth are among the most globally connected in Central Asia. Their value orientations likely reflect the influence of global cultural currents that prize individual achievement, entrepreneurship, and skepticism of authority. At the same time, the older generation may hold more tightly to local traditional values or the “neotraditional” revival (such as heightened religiosity or community-centric attitudes) as a counter-response to rapid change. The push and pull between global modernity and local tradition creates a generational gap in values. Our findings of youth leaning secular and individual, versus elders remaining somewhat more religious and communal, exemplify this tension that globalization theory highlights.

The political-economic context of Kazakhstan also shapes these value differences in crucial ways. The past three decades saw not only urbanization (over 60% urban by 2024) and economic growth, but also the consolidation of an authoritarian-but-stable political order. Urbanization and digitalization have given young people access to diverse ideas and networks, enabling them to form identities less dependent on immediate communities. A city-bred, internet-savvy youth is more likely to question traditional authorities and seek self-expression – indeed, our youth respondents’ low trust in traditional media and officials is indicative of a digitally informed skepticism. Meanwhile, rising inequality and changing social policies may have bifurcated youth experiences: some urban middle-class youth feel materially secure and thus focus on postmaterialist goals, while others aware of inequality may adopt a cynical outlook, disengaging from institutions they perceive as corrupt or unresponsive.

Junisbai and Junisbai (2020) found that the new generation in Kazakhstan is more accepting of inequality and expects less from the state, which dovetails with our observation that youth do not look to government or collective structures for support, instead emphasizing personal responsibility. This could be a reaction to a political context where the state, despite various youth programs and rhetoric, is not seen as empowering young people’s voices. Kazakhstan’s government has launched initiatives (e.g., the Youth of Kazakhstan strategy, and social programs in education and employment), aiming to integrate youth and address their needs. However, our findings suggest a mismatch between official narratives and youth’s lived values: young people seem to be charting their own course, driven by global influences and their perceptions of limited institutional efficacy. For example, while the state’s rhetoric under programs like Rukhani Zhangyru extols traditional values and loyalty to the nation’s development, youth in our survey demonstrate lukewarm attachment to formal patriotic or collective values (implied by low institutional trust and lower emphasis on communal activities). Instead, they focus on personal advancement (education, career aspirations albeit on their own terms) and private life (family, lifestyle). This does not mean Kazakhstani youth are anti-social or devoid of morals – rather, their social consciousness is being expressed in new, more individualized ways. They might engage through informal networks, social media, or entrepreneurial ventures rather than through the established institutional pathways their parents respected.

Crucially, these generational differences have important sociological and practical implications. The widening gap in values and trust between youth and the rest of society could lead to intergenerational frictions or a sense of alienation. For instance, if young people are less committed to traditional workplace norms, they may struggle with or seek to transform the organizational culture once they enter full-time employment. Their low trust in government and political parties may translate into apathy or unconventional forms of political expression, potentially weakening conventional civic engagement while increasing the volatility of public opinion. The fact that youth highly value family but not broader friendships might mean that social capital is becoming more narrowly concentrated – strong bonds in the family but weak bridging ties in the community – which Robert Putnam’s theory suggests can erode the fabric of civil society. Additionally, the secular and self-reliant mindset of youth may clash with the expectations of elder family members or community leaders who anticipate deference to tradition and authority. In Kazakhstan, where respect for elders and communal cohesion has been a key societal glue, such a shift could challenge the transmission of cultural norms and complicate the integration of youth into adult society. As some researchers have warned, if youth increasingly orient toward individualistic values at the expense of collective responsibilities, it could pose risks for social cohesion and public loyalty (Putnam, 2000; Welzel and Inglehart, 2005). Our findings indeed hint at nascent forms of what one might call value-based generational segmentation, where the young carve out a moral universe somewhat apart from that of their parents.

Nonetheless, understanding these differences also offers an opportunity: policy-makers and educators can proactively bridge this gap by adapting to the evolving value landscape. The Kazakhstani government and civil society organizations could use the common ground (family values and desire for well-being) as a starting point to engage youth on other issues. For example, framing public programs or national narratives in terms of how they benefit families and personal development might resonate more with youth. Efforts to increase youth trust in institutions might focus on transparency, accountability, and youth inclusion in decision-making, to show young citizens that their individual voices matter in the collective arena. Educational curricula and community initiatives could address the apparent deficit in social capital by fostering volunteerism, peer networking, and open dialog between generations, thereby expanding youths’ circle of trust beyond the family without forcing a return to old forms of authority. Moreover, recognizing that youth place high value on self-realization, there should be support for entrepreneurship, creative industries, and other avenues that allow personal initiative – these can channel youth’s individualistic energies into productive contributions that also serve society. In sum, the differences we observe are not merely a curiosity of survey data; they signal where Kazakhstani society might be headed and where attention is needed to ensure social integration. As the country continues to modernize, the challenge will be to accommodate growing individualism and secular orientations among youth while maintaining social cohesion and intergenerational solidarity. This calls for an adaptive approach to cultural and social development policy, one that acknowledges youth as agents of change with distinct values, rather than simply bearers of problems. By integrating the fresh perspectives of the young generation with the wisdom and experience of the older generation, Kazakhstan can strive for a value consensus that leverages the best of both worlds – the innovative spirit of youth and the enduring strengths of its traditions.

In conclusion, the value profile of Kazakhstani youth is characterized by a mix of alignment and divergence with broader societal values. Family and basic well-being priorities create a common foundation between youth and their elders, even as significant gaps in areas like work, religion, and trust point to a transformative shift in progress. These findings reinforce and extend theoretical expectations from modernization and globalization theories: we are witnessing the early stages of a cultural shift where post-materialist and individualist values gain ground within a traditionally collectivist society. While we have interpreted these patterns through established sociological lenses (secularization, individualization, social capital theories), a fuller exploration of these concepts was beyond the scope of our data and was not extensively covered in the introductory sections. Future research and an expanded literature review should integrate these theoretical perspectives more deeply to contextualize the ongoing changes. Nevertheless, our discussion highlights the essential point that Kazakhstani youth sit “between traditions and globalization”: they uphold certain enduring values shared with their community, but they are also pioneers of new value priorities that will shape Kazakhstan’s social landscape in the years to come. Recognizing both the continuities and changes in youth values is vital for academics and policymakers alike as they work to foster mutual understanding and guide the country’s development in a way that is inclusive of the next generation’s aspirations.

## Conclusion

This study confirms that Kazakhstan’s younger generation stands at a crossroads between enduring traditions and the forces of modernity. The value profile of Kazakhstani youth is characterized by both alignment with and divergence from the values of the broader population. On one hand, young people continue to uphold fundamental traditional values—family solidarity and basic material security remain central, much as they do for older generations, providing a common foundation across age groups. On the other hand, clear generational gaps have emerged in domains such as work, religion, and social trust, where youth attitudes differ markedly from those of their elders. These divergences signal a transformative shift: individualistic and post-materialist values are gradually gaining ground within a society long anchored in collectivist norms. In line with classic modernization theory, as Kazakhstan has urbanized and developed, younger cohorts exhibit a growing emphasis on self-expression, personal ambition, and secular outlooks, moving away from some of the survival-oriented and conformist values characteristic of the previous generation. In short, Kazakhstani youth today occupy an intermediate space “between traditions and globalization,” simultaneously preserving core cultural values and pioneering new priorities that will shape the country’s social landscape in the years to come. Recognizing both the continuities and changes in youth values is vital for sociologists and policymakers seeking to understand and guide Kazakhstan’s development in an inclusive manner.

Beyond these broad value shifts, the findings shed light on several specific aspects of youth attitudes and well-being. Subjective well-being: Young respondents report higher overall life satisfaction than the general adult population, reflecting a relatively optimistic outlook on their current lives and future prospects. This heightened optimism suggests that many youths feel empowered by the opportunities of modern Kazakhstani society. However, it is noteworthy that a significant minority of young people express dissatisfaction with their life circumstances, a reminder that not all are equally benefiting from recent social and economic changes. These discontented voices underscore persistent inequalities and the need for greater attention to the unmet expectations of some youth. Health perceptions: Kazakhstani youth also tend to rate their personal health more favorably than older cohorts, which is expected given their age and greater awareness of healthy lifestyles. Yet, many young people face practical constraints—limited financial resources and economic uncertainties can impede their ability to fully realize their ambitions. This tension between high aspirations and material challenges illustrates the complexity of the youth experience: even as values shift toward self-realization, structural factors may limit the fulfillment of those goals.

In the civic and environmental domains, the younger generation displays a mix of encouraging engagement and areas for growth. Environmental attitudes: Youth exhibit a nuanced stance toward environmental issues. They generally recognize the importance of protecting the environment and show willingness to balance ecological concerns with economic interests. At the same time, the survey revealed a gap in awareness of global environmental challenges among young people. This suggests that while local environmental consciousness is present, broader global issues like climate change may not be fully on their radar. Enhancing environmental education and awareness programs could bridge this gap, empowering youth to participate more actively in global sustainability initiatives. Civic engagement: Meanwhile, Kazakhstani youth demonstrate a strong commitment to their communities through high rates of participation in volunteer activities. This willingness to volunteer reflects a sense of civic responsibility and social engagement at the grassroots level. It points to considerable potential for the development of civil society: young people are not disengaged or apathetic, but rather are ready to contribute to social causes given the opportunity. Supporting youth-led initiatives and volunteer organizations could thus harness this energy and further strengthen civic bonds. Taken together, these patterns portray a generation that is optimistic and civically minded, yet navigating the practical realities of a rapidly changing society.

While the results provide valuable insights, it is important to acknowledge the study’s limitations. Scope of sample: First, the youth survey sample may not fully represent the diversity of Kazakhstan’s young population, especially in terms of regional, ethnic, and socio-economic subgroups; some voices and nuances may not have been captured. Comparative constraints: Second, the comparison with the 2018 World Values Survey data, though illuminating, is constrained by methodological differences in survey design and implementation. Caution is warranted when directly comparing percentages or drawing causal inferences, as some discrepancies might arise from these differing methodologies rather than true change. Temporal context: Third, this research offers a snapshot of youth values at one moment in time. Value orientations are dynamic and can evolve quickly in response to ongoing economic, political, and cultural developments. Therefore, longitudinal data would be necessary to track how the trends identified here continue to unfold and whether the observed generational gaps widen or converge in the future.

Building on these findings, several directions for future research and practice are recommended. To deepen our understanding of youth values, future studies should include broader and more diverse samples of young people across various regions and social backgrounds, ensuring that smaller or marginalized groups are represented. Longitudinal research would be particularly beneficial: tracking the same cohorts over time can illuminate how youth value priorities change as they age and as Kazakhstan itself changes. It would also be valuable to integrate interdisciplinary perspectives by examining economic, cultural, and psychological factors that influence value formation—such as the impact of labor market conditions, family upbringing, or individual life experiences on youths’ priorities. Additionally, given the rising importance of digital life, research should investigate the role of the online environment and social media in shaping values and attitudes. Kazakhstan’s youth are among the most digitally connected in the region, and their exposure to global information flows could significantly affect their worldview, from social norms to political engagement. Understanding these influences will provide a more comprehensive picture of how globalization is transmitted at the individual level.

Finally, the insights from this study carry important implications for policy and practice. Policymakers, educators, and community leaders should take into account the evolving value orientations of Kazakhstani youth when designing programs and interventions. Youth policies and educational curricula that acknowledge young people’s heightened desire for personal growth, autonomy, and civic involvement are more likely to resonate and be effective. For example, employment and entrepreneurship programs can be tailored to balance the desire for self-realization with practical skill-building, and civic education initiatives can leverage the existing enthusiasm for volunteerism to foster greater social trust and participation. Likewise, addressing the concerns of dissatisfied youth—through targeted social support, inclusive decision-making, and open dialog—could mitigate potential intergenerational frictions and strengthen social cohesion. By aligning national development strategies with the priorities of the emerging generation, Kazakhstan can better facilitate the social integration of its youth and harness their potential as agents of positive change. In sum, acknowledging and engaging with the values of young Kazakhstanis will not only support their personal development but also contribute to the sustainable development of the country as a whole.

## Data Availability

The original contributions presented in the study are included in the article/supplementary material, further inquiries can be directed to the corresponding author.
